# Study of Ovarian Damage in Piglets in an Experimental Model of Neonatal Asphyxia

**DOI:** 10.3390/children12030371

**Published:** 2025-03-17

**Authors:** Efstathia-Danai Bikouli, Rozeta Sokou, Monica Piras, Abraham Pouliakis, Eleftheria Karampela, Styliani Paliatsiou, Paraskevi Volaki, Gavino Faa, Theodoros Xanthos, Christos Salakos, Nicoletta M. Iacovidou

**Affiliations:** 1 Department of Neonatology, Medical School, Aretaieio Hospital, National and Kapodistrian University of Athens, 11528 Athens, Greece; sokourozeta@yahoo.gr (R.S.); stpaliatsiou@yahoo.gr (S.P.); v.volaki@hotmail.com (P.V.); 2 Neonatal Intensive Care Unit, General and Maternity Hospital “Helena Venizelou”, 11521 Athens, Greece; 3 Department of Medical Sciences and Public Health, University of Cagliari, 09124 Cagliari, Italy; monica.piras@unica.it (M.P.); gavinofaa@gmail.com (G.F.); 4 Second Department of Pathology, “ATTIKON” University Hospital, Medical School, National and Kapodistrian University of Athens, 12462 Athens, Greece; apou1967@gmail.com; 5 Experimental, Educational and Research Center, ELPEN Pharmaceutical, 19009 Pikermi, Greece; ekarampela@elpen.gr; 6 Department of Biology, College of Science and Technology, Temple University, Philadelphia, PA 19122, USA; 7 School of Health Sciences, University of West Attica, 12243 Athens, Greece; theodorosxanthos@yahoo.com; 8 Medical School, National and Kapodistrian University of Athens, 11527 Athens, Greece; csalakos@otenet.gr

**Keywords:** perinatal asphyxia, neonates, ovaries, female reproduction, resuscitation, histology

## Abstract

**Background/Objectives**: Perinatal asphyxia constitutes a major complication of the perinatal period with well-described effects on multiple organs and systems of the neonate; its impact, though, on the ovaries is hardly known. The objective of the present study was to investigate potential histological alterations of the ovaries in an animal model of perinatal asphyxia with or without resuscitation. **Methods**: This was a prospective, randomized animal study; 26 female Large White/Landrace piglets, aged 1–4 days, were the study subjects and were randomly allocated in 3 groups. In Group A (control), the animals had their ovaries surgically removed without any manipulation other than the basic preparation and mechanical ventilation. The other 2 groups, B (asphyxia) and C (asphyxia/resuscitation), underwent asphyxia until bradycardia and/or severe hypotension occurred. At the hemodynamic compromise, animals in group B had their ovaries surgically removed, while animals in group C were resuscitated. Following return of spontaneous circulation (ROSC), the latter were left for 30 min to stabilize and subsequently had their ovaries surgically removed. The ovarian tissues were assessed by the pathologists for the presence of apoptosis, balloon cells, vacuolated oocytes, and hyperplasia of the stroma. The histological parameters were graded from 0 (absence) to 3 (abundant presence). **Results**: The presence of balloon cells and apoptosis was found to be more prominent in the ovaries of animals in groups B and C, compared to that of the control group at a statistically significant degree (*p* = 0.0487 and *p* = 0.036, respectively). A significant differentiation in balloon cell presence was observed in cases with higher grading (2–3) in the asphyxia group (with or without resuscitation) (*p* value: 0.0214, OR: 9, 95% CI: 1.39–58.4). Although no statistically significant difference was noted regarding the other 2 histological parameters that were studied, there was a marked negative correlation between the duration of asphyxia and grade of vacuoles in oocytes when the potential effect of the duration of asphyxia or resuscitation on the histological findings was investigated (r = −0.54, *p* = 0.039). **Conclusions**: We aimed at investigating the potential effect on the neonatal ovaries in our animal model of perinatal asphyxia. Given that the presence of apoptosis and balloon cells was more prominent in cases of asphyxia, it can be speculated that perinatal asphyxia might have an impact on the neonatal ovaries in addition to the other, better-studied systemic effects. More research is needed in order to clarify the potential effect of perinatal asphyxia on the ovaries.

## 1. Introduction

Perinatal asphyxia is a major incidence of the perinatal period with high morbidity, often associated with severe adverse outcomes for the baby’s health, both in the short- and the long-term. Defined as “a state of severe hypoxia affecting the offspring around the time of birth, as a result of several maternal conditions and labor complications”, perinatal asphyxia accounts for approximately 900,000 deaths per year, according to the World Health Organization (WHO), while affecting around 4 million neonates per year worldwide [1].

The reported incidence of severe perinatal asphyxia varies in different settings. It is estimated to be approximately 1/1000 live births in resource-rich countries and higher in resource-limited countries, where it is estimated at around 5–10/1000 live births, according to data from hospital settings [2].

Although the most prominent and well-studied sequel of perinatal asphyxia is the development of hypoxic ischemic encephalopathy (HIE), with an estimated incidence at approximately 2.5 per 1000 live births [3], oxygen deprivation and subsequent biochemical alterations are known to affect almost all of the human organs and tissues. In order for the brain, heart, and adrenal glands to be well perfused, the circulation is redistributed towards these vital organs at the cost of the rest of the tissues, thus leading to hypoxia and secondary tissue damage [3]. The effects of perinatal asphyxia on the liver, kidneys, cardiac function, and gastrointestinal tract have already been well studied [3]. Therefore, the term “multiple organ failure” (MOF) or “multiorgan dysfunction” (MOD) can also be applied in perinatal asphyxia and its complications.

Given the systematic involvement and pathophysiological basis of MOD, the speculation that ovaries may also be affected by perinatal asphyxia and alterations in their histology and function may occur in cases of perinatal asphyxia seems to be reasonable. However, the fact that ovarian function starts at puberty makes the investigation of the existence of such alterations challenging, since very few studies have looked into this issue.

Uncovering the potential effect of perinatal asphyxia on the ovaries may disclose several pathophysiological pathways of either the reproductive system or different systems and organs. The significance of ovarian function for females is well described and proven in several fields besides the reproductive function. Cardiovascular system health, bone mass, and skeletal integrity are only a few of the areas in which normal ovarian function is important and beneficial. Therefore, a question arises whether the indirect effect that perinatal asphyxia may have on other systems through potential disruption of ovarian structure and function.

A very interesting observation from experimental and human studies is that several outcomes affecting development appear to be gender-dependent. More specifically, HIE has a more unfavorable outcome in males compared to female infants, while male infants have increased mortality rates, respiratory distress syndrome, and long-term developmental disorders, thus suggesting that gender seems to be an important factor for the severity and clinical outcome of hypoxia [4]. The term “male disadvantage” was introduced with regards to the perinatal period and refers to the fact that male fetuses were at a greater risk of adverse pregnancy outcomes, had increased rates of NICU admission, had an increased risk of renal and neurological impairment, and were more vulnerable to the effects of hypoxia [5]. The sex of the fetus is also associated with differences during in utero development, as well as with differential response to several drugs [6].

There are also indications that the timing of exposure to hypoxia may affect the presentation of differential neurodevelopmental consequences in male and female infants.

The pathophysiology and mechanism of these gender-dependent manifestations are not clarified, but gonads supposedly play an important role. Therefore, a potential effect of perinatal asphyxia on the ovarian structure and function could further disrupt homeostatic mechanisms and lead to altered programming of different organs and systems.

The primary objective of our study was to investigate and unveil potential pathology deviations of the histology of neonatal piglet ovaries in perinatal asphyxia. In addition to this, we aimed at looking into the potential reversibility of these alterations in case of successful resuscitation. The reason why the swine were selected as species for the experimental process of our study is mainly attributed to the fact that the ovaries and follicular development of pigs have been shown to bear a close resemblance to the human ovaries [7], while swine are considered to be species quite similar to humans with reference to both anatomy and physiology in general [8]. It can therefore be expected that potential findings occurring from our research in piglet ovarian tissue can reflect similar alterations in human ovaries.

Since pathology alterations can relate to a significant degree to pathophysiological and functional deviations, the potential findings of our study could prove useful in further investigating and unveiling the impact of perinatal asphyxia on the ovarian function. Therefore, the potential clinical impact of our study would be to attempt to bridge the critical gap in understanding the long-term consequences of perinatal asphyxia on female reproductive health. Taking into consideration the complicated and multisystemic role of the ovaries in endocrine regulation and overall systemic homeostasis, any disruption at this stage could have far-reaching implications, potentially affecting fertility, hormonal balance, and metabolic health later in life.

## 2. Materials and Methods

This was a prospective, randomized animal study.

The study protocol was approved by the Greek General Directorate of Veterinary Services (Approval number: 1553/05/04/2018) and was conducted in accordance with the Greek legislation and the European Parliament Directives.

### 2.1. Animal Preparation

In total, 33 female Landrace/Large White piglets, aged 1–4 days old, with a weight of 1300–2400 g were used as the study subjects. All animals were supplied by the same breeder (Validakis, registered breeder, Koropi, Attica, Greece), and they were transferred to the experimental facility (ELPEN’s Experimental, Research and Training Centre, European Ref No. EL 09 BIO 03) on the day of experimentation. Prior to the commencement of the experiment, all animals were examined by a veterinarian who confirmed their normal health status. Animals were treated in compliance with the Guide for the Care and Use of Laboratory Animals.

All the animals were initially sedated with intramuscular administration of ketamine hydrochloride (10 mg/kg) (Imalgène, Merial Laboratorios SA, Lyon, France), midazolam (0.5 mg/kg) (Dormicum, Roche, Athens, Greece), and atropine sulfate (0.01 mg/kg) (Atropine sulfate, Demo, Athens, Greece), as previously described [9]. Following that, the animals were transferred to the operating table, and their lateral auricular veins were catheterized. Subsequently, the animals were anesthetized via intravascular administration of propofol (1 mg/kg) (Diprivan 1% *w*/*v*; Astra Zeneca, Luton, UK) and fentanyl (10 μg/kg) (Janssen Pharmaceutica, Beerse, Belgium) and intubated with a Portex cuffed endotracheal tube size 4 (Portex, 4.0 mm ID; Mallinckrodt Medical, Athlone, Ireland) using a curved blade laryngoscope. The correct placement of the endotracheal tube was confirmed with auscultation.

During the experimental procedure, the piglets were immobilized in a supine position and remained under continuous heart rate, ECG (leads I, II, III, avR, avL, and avF) (Mennen Medical, Envoy; Papapostolou, Athens, Greece), and oxygen saturation monitoring through a SpO_2_ sensor placed on their tongues. The animals during the whole experiment lay on thermal mattresses under radiant warmers for thermoprotection; their temperature was monitored using a rectal thermometer aiming at maintaining a temperature of 38°C +/−1 °C.

Bolus doses of fentanyl (20 μg/kg) and cis-atracurium (0.15 mg/kg) (Nimbex 2 mg/mL; GlaxoSmithKline, Athens, Greece) were administered intravenously to the piglets, and following that they were mechanically ventilated with initial settings of tidal volume of 10–15 mL/kg, peak inspiratory pressure of 19 cmH_2_O, and respiratory rate of 30–40/min, which were further adjusted according to the blood gases and the end-tidal carbon dioxide (ETCO_2_), whose target values ranged between 35 and 45 mmHg. Arterial blood gases were measured on a blood–gas analyzer (IRMA SL Blood Analysis System, part 436301; Diametrics Medical Inc, Roseville, MN, USA). Finally, oxygen supply was regulated in order to maintain SpO_2_ levels of 90–95%.

Throughout the experimental procedure, the animals remained under continuous intravenous infusion of NaCl 0.9% at an infusion rate of 10 mL/kg/h and D/W 5% at an infusion rate of 5 mL/kg/h in order to avoid dehydration and hypoglycemia, respectively. Sedation and anesthesia were maintained with continuous intravenous propofol infusion (8–10 mg/kg/h) and bolus intravenous administration of fentanyl (10 μg/kg) and cis-atracurium (0.15 mg/kg) as indicated.

At the last part of the animal preparation, the animals’ common carotid and internal jugular veins were surgically revealed and catheterized with 3.5 Fr central catheters. The catheters were connected to electronic pressure transducers (3.5 Fr, USCI CR, Bart; Papapostolou) for continuous monitoring of systolic, diastolic, and mean arterial pressure and right atrial pressure.

Following the catheter insertion and prior to the commencement of the experiment, all animals were left to stabilize for 30 min. Stabilization was defined based on the hemodynamic parameters as follows:

⚬ Heart rate: 120–180 beats/min.
⚬ Systolic arterial blood pressure: 70–90 mmHg.
⚬ Mean arterial blood pressure: 60–80 mmHg.
⚬ Diastolic arterial blood pressure: 40–60 mmHg.
⚬ Central venous pressure: 2–8 mm Hg.
⚬ SpO_2_: 90–95%.

Following stabilization, blood and urine samples were obtained from all the piglets, and the main experiment commenced.

### 2.2. Experimental Protocol

The study animals were randomly allocated into 3 groups via closed envelopes as follows:

- Group A (12 piglets): control.
- Group B (11 piglets): perinatal asphyxia with no resuscitation.
- Group C (10 piglets): perinatal asphyxia with resuscitation.

Group A piglets remained under sedation and mechanical ventilation for one hour following stabilization, after which blood and urine samples were obtained. The animals’ ovaries were surgically removed, and the piglets were euthanized with intravenous administration of pentovarvital (200 mg/kg) (Dolethal, Vetoquinol SA, Lure, France).

In groups B and C animals, their endotracheal tube was occluded, and intravenous infusion of propofol was ceased. The endpoint of asphyxia was the manifestation of hemodynamic compromise, defined as either bradycardia (HR < 60/min) or severe hypotension (MAP < 15 mmHg). The duration of the time elapsed from occlusion of the endotracheal tube to hemodynamic compromise was recorded in all animals, and additional blood and urine samples were obtained. Group B animals had their ovaries surgically removed, and this was the experiment’s endpoint for them.

After the manifestation of asphyxia and the confirmation of hypoxemia in arterial blood gases, animals in group C piglets were resuscitated according to the ILCOR 2015 guidelines [10]. Ventilation breaths were administered via Neopuff™ Infant Resuscitator (Fisher & Paykel Healthcare, Auckland, New Zealand) set at a Peak Inspiratory Pressure (PIP) of 30 cmH_2_O and a Positive End-Expiratory Pressure (PEEP) of 5 cmH_2_O. Gas flow supply was set at 8 L/min.

Hemodynamic parameters were continuously monitored throughout the experiments, and the time periods of asphyxia, resuscitation, return of spontaneous circulation (ROSC), and stabilization were recorded, as were the type and duration of resuscitation applied in each case and the administration of adrenaline, if indicated. ROSC was defined as the return of the hemodynamic parameters to the levels of the stabilization period with a deviation of +/− 10%. The endpoints of the experiment in group C piglets were defined as either persistent asystole despite sufficient resuscitation efforts for 10 min or return of spontaneous circulation (ROSC) and restoration of the hemodynamic parameters as previously mentioned.

Arterial blood gases were obtained at ROSC, and following that group C piglets that, were successfully resuscitated remained ventilated under sedation for 30 min with a target MAP of 50 mmHg. After that period of stabilization, blood and urine samples and arterial blood gases were obtained in order to confirm the animals’ steady state. Then, the ovaries were surgically removed, and the animals were humanely euthanized with intravenous bolus administration of pentovarvital (200 mg/kg).

The removal of the ovaries in all animals was performed by the same members of the experimental team, using the same technique and under the administration of the same drugs, in order for the whole procedure to be similar among the animals, so as to minimize bias. The reproductive system in female pigs is located dorsal to the intestines in the pelvic cavity. In pigs, each ovary is attached to a highly coiled uterine horn (similar to a human’s fallopian tubes).

A ventral midline incision was performed at the level of the two most caudal teats. Following incision of the linea alba and entering the abdomen, the uterus and ovaries were traced through visual and digital inspection. Subsequently, both of the ovaries as well as the uterus were removed en bloc and immediately placed in formalin solution (4% concentration).

Strict adherence to resuscitation and post-resuscitation guidelines facilitated the minimization of treatment bias. Resuscitation was performed by qualified and sufficiently trained providers.

**Figure 1:**
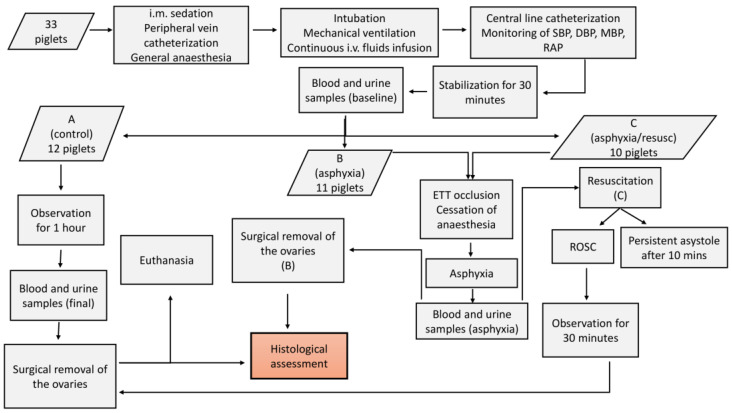
Flowchart presenting the experimental process and steps of the study protocol. A, B, and C refer to the categories of the animals. (B) and (C) are used to indicate that the respective steps apply to this specific category that is mentioned. i.m.: intramuscular, i.v.: intravascular, SBP: systolic blood pressure, DBP: diastolic blood pressure, MBP: mean blood pressure, RAP: right atrial pressure, ETT: endotracheal tube, ROSC: return of spontaneous circulation.

The experimental protocol and overall procedures of our study are briefly presented in Figure 1.

### 2.3. Histological Preparation and Evaluation

Following removal, the ovaries were immediately fixed in formalin solution (10% formaldehyde diluted to 4%) (formaldehyde solution 4%, buffered with phosphate buffer, pH 6.9, Sigma-Aldrich, Taufkirchen, Germany) and preserved at room temperature. The samples were subsequently processed by dehydration in increasing alcohol scale (50%, 70%, 80%, 90%, and 100%), followed by clarification and inclusion in paraffin. Clarification was performed with the gradual replacement of the transition medium, specifically xylene. Serial sections were then prepared for each sample, the first of which were 3 µm thick using the rotary microtome. The sections were rehydrated, paraffined, embedded on glass slides, and stained with standard hematoxylin and eosin staining for histomorphological evaluation. They were analyzed under a light microscope in a blinded, randomly numbered way.

Then, 20 sections were prepared from every ovarian tissue and further assessed, and 5 sectional areas were observed from each slide.

We looked for the presence of balloon cells, vacuoles in oocytes, stromal cells, and apoptosis.

All parameters were described and presented for each sample using a four-level semiquantitative scale ranging from 0 to 3 (0: none, 1: mild, 2: moderate, and 3: severe). The grading of each parameter was defined based on the percentage of extent of its presence over 100 sectional areas, and the grades referring to each sample and histological parameter were given by the pathologists who assessed the tissue sections.

The refined grading system that was used provides a detailed assessment of the degree of lesion severity in ovarian tissue, facilitating more accurate characterization and interpretation of histological changes. The scoring system for ovarian lesions is outlined as follows:

Grade 0: No discernible evidence of the respective lesions. This grade indicates a normal or unaffected tissue appearance, where both balloon cell formation and apoptosis are absent or too minimal to be detected microscopically.

Grade 1: Occasional, scattered presence of respective parameters. At this stage, balloon cells may be present in isolated areas, along with a few apoptotic cells scattered throughout the tissue. These findings suggest an early or mild stage of lesion development, where the alterations are limited and not widespread.

Grade 2: Extensive presence of respective lesions. Here, the balloon cells and apoptotic bodies are more diffusely distributed throughout the tissue, reflecting a moderate level of cellular degeneration. The involvement of a larger portion of the tissue suggests a more advanced stage of lesion formation, though it may still be reversible at this point.

Grade 3: Confluent presence of respective lesions. More specifically, a confluence of balloon cells, undergoing cytolysis, resulting in the formation of pseudocystic spaces, along with the aggregation of adjacent apoptotic cells. This represents the most severe stage of lesion progression, where balloon cells begin to merge, undergoing cytolytic processes that culminate in the formation of pseudocystic spaces. Simultaneously, apoptotic cells cluster together, further indicating extensive cellular damage. This stage suggests irreversible tissue injury and significant morphological disruption, often associated with advanced pathological processes.

This grading system provides a clear and systematic approach to evaluating the severity of ovarian lesions, facilitating better correlation with clinical outcomes and enhancing the accuracy of histopathological assessments.

### 2.4. Sample Size Calculation

Based on a study by Ural et al. [11], who looked into the biochemical, histopathological, and immunohistochemical alterations in a rat model of ovarian ischemia and ischemia/reperfusion and the potential effect of thymoquinone administration, histopathological scores were expected to have a standard deviation (SD) of 0.5. In order for the study design to detect a difference of 1.5 × SD = 0.75 units with a power of 80% and at a level alpha = 0.017 (0.05/3 in view of Bonferroni correction), the total number of piglets required for the experiment was estimated at 33. The study power calculation was based on the G-power 3.1.9.2 program.

Therefore, the initial study population of our study was expected to be 33 animals, and this was further augmented by 2 more animals (allocated to groups A and B). However, during the initial preparation manipulations, 2 of the animals unfortunately passed away. These animals had been allocated to groups B and C.

Following the completion of the experiments and prior to the statistical processing of the results, 7 samples were excluded from our study, mainly because of technical reasons during the experimental procedure that hampered the accomplishment of the experiments in accordance with the study protocol. More specifically, one animal in the control group (A) was excluded because of difficulty in ventilating it, and that would not allow for the animal to be considered control (as was also indicated by the arterial blood gases obtained during the steady state), 2 animals from the asphyxia group (B) were excluded because of ventilator malfunction leading to inability to ventilate, and carotid artery hemorrhage during the insertion of the central catheters, respectively, and 4 animals that had been allocated to the asphyxia/resuscitation group (C) were excluded as 3 of them were unable to obtain and maintain a steady “baseline” state, while in the fourth one, intrabdominal hemorrhage occurred that led to cardiovascular compromise.

Following the exclusion of the abovementioned samples, the study population consisted of 26 animals in total, and their group allocation was as follows:

Group A (control): 11 animals.
Group B (asphyxia): 9 animals.
Group C (asphyxia with resuscitation): 6 animals.

The grading and descriptions of each parameter referring to each sample were presented on a spreadsheet, and the data were classified together depending on the category of the sample (A, B, or C).

### 2.5. Statistical Analysis

The statistical analysis was performed with the SAS 9.4 software for Windows (SAS Institute Inc., Cary, NC, USA) [12,13]. In terms of descriptive statistics, there is a full presentation of data regarding the mean value and median as well as measures of variability (i.e., standard deviation and first and third quartiles). As for the qualitative variants, we presented the frequencies and relative percentages. Moreover, given that the numeric variables are expected to receive a limited range of values (0 to 3, integers), they are also presented as frequencies and percentages. In view of the small number of cases, the comparisons between the different groups were performed with the Fisher exact test. In the case of numeric variable analysis, the *t*-test or ANOVA test (for two or more groups, respectively) was utilized for normal distribution, while in the absence of normal distribution, we performed the Mann–Whitney and Kruskal–Wallis tests for comparisons between two or more groups, respectively. Statistical significance was set at a p value lower than 0.05 for all tests.

## 3. Results

### Histopathological Results

Following exclusion of the samples that did not meet the study criteria, the study population consisted of 26 female piglet neonates that had been randomly allocated into 3 groups (group A/control: 11 piglets, group B/asphyxia: 9 piglets, group C/asphyxia& resuscitation: 6 piglets). There were no statistically significant differences between the groups with regards to the animals’ demographic characteristics (age and birthweight-BW) and baseline hemodynamic characteristics (SpO_2_, heart rate, mean arterial pressure, lactate, and pH). The respective data and p-values are presented in Table 1. Therefore, no confounding factor referring to the animals’ baseline condition would be anticipated.

**Figure 2:**
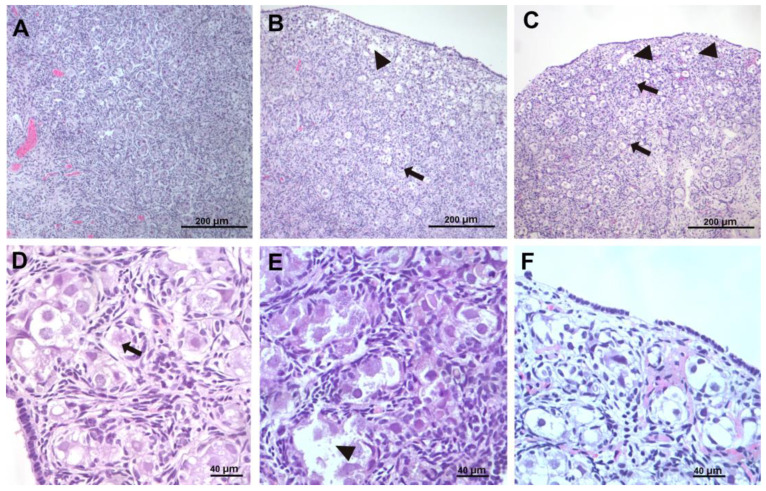
(**A**,**D**) Normal sample (control group). Small vacuoles in the oocytes (normal), arrow. (**B**,**E**) sample from group B animal (asphyxia) showing diffuse damage. Arrow points to apoptotic cell, and arrowheads point to balloon cells. (**C**,**F**) sample from group C animal (asphyxia and resuscitation). Arrow points at follicles undergoing apoptosis in the deep cortex; arrowheads point to edema in the superficial cortex and to ballooning follicles undergoing lytic necrosis. (**A**–**C**) 10× magnification, (**D**–**F**) 40× magnification.

The histological samples that were obtained according to the protocol were assessed in a blinded way, and the descriptions and grades for each parameter and sample were provided by the pathologists. Indicative pictures from the histological samples of each category are provided in Figure 2.

The aggregated data per group and histological parameter are shown in Table 2.

**Figure 3:**
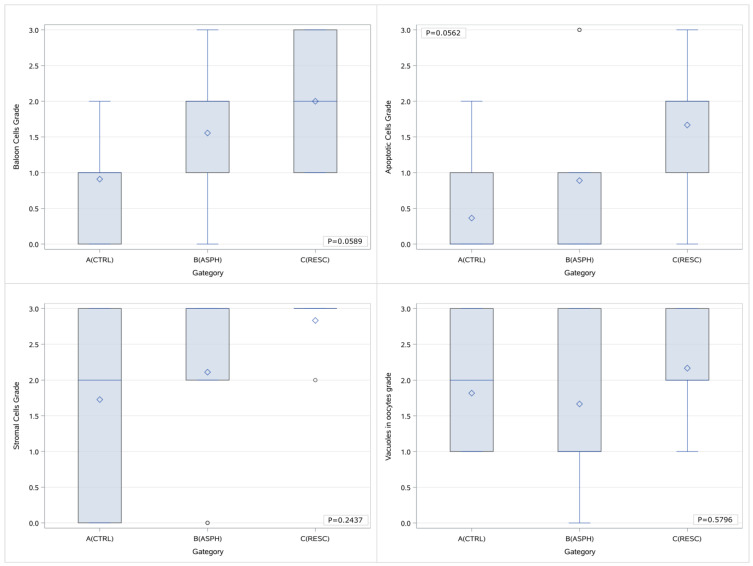
Box & Whisker plots of the histological parameters grading per study group and p values of the comparisons (concurrent between all groups). A (CTRL): control group, B (ASPH): asphyxia group, and C (RESC): asphyxia and resuscitation group. In each diagram, the box limits indicate the lower (1^st^) and higher (3^rd^) quartiles (Q1 & Q3 respectively), horizontal lines within the boxes indicate median value, while the limits of the whiskers indicate minimum and maximum values after excluding outliers. Mean values are visible as diamond symbols and circles outside the whisker areas indicate outliers.

The Kruskal–Wallis test was used for the comparison of the numerical values of the grading for the three groups of animals concomitantly. No statistically significant difference was noted between the three groups with regards to the histological parameters that were looked into when the grading is processed as a number. However, there is a marked differentiation trend in the presence of balloon cells and apoptosis. The results for each comparison are presented in a Box & Whisker diagram, and the diagrams are depicted in Figure 3.

Comparisons were also performed using the Fisher exact tests and considering the four different gradings as separate categories. Although the presence of a differentiation trend was noted between the three groups regarding the apoptotic cell grade, no statistically significant difference was highlighted with reference to any of the histological parameters that were investigated.

An additional comparison between the 3 groups that was attempted during the statistical processing involved classifying the histological gradings in two distinct categories depending on the severity of the lesions. More specifically, in an attempt to highlight the potential existence of histological lesions, the first category referred to the mild presence of the histological parameters (gradings 0 and 1), while the other included the more profound cases of histological findings (gradings 2 and 3). Given the absence of numerical variables and the limited number of cases, the statistical test that was performed in this case was the Fisher exact. The analysis of the results highlighted the presence of a marked difference in the balloon cell grade as well as the apoptotic cell grade. More specifically, with regards to balloon cells, the percentage of cases with a low grade (0–1) in the control group (A) was 81.82%, whereas in groups B and C, this is reversed, and the percentage of cases with a high grade (2–3) was 66.7% (*p* value: 0.04871). As far as the apoptotic cells are concerned, the percentage of low grades (0–1) in the control group was 90.9%, while the respective percentages in groups B and C were 77.8% and 33.3% (*p* = 0.0364). There was no differentiation in the cases of stromal cells and vacuoles in oocytes. The data obtained through the classification we mentioned above are presented in Table 3.

**Figure 4:**
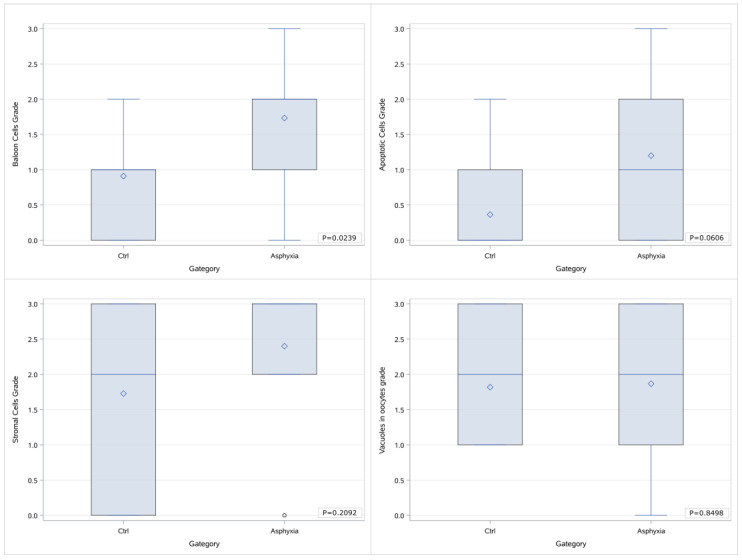
Box & Whisker diagrams of the grading of histological parameters in 2 groups of samples (control vs. asphyxia) and *p* values of the comparisons.

We attempted further categorizations by grouping the samples as asphyxiated (B & C) versus nonasphyxiated (A). As presented in the Box & Whisker diagrams below (Figure 4), when processing the grade as a numerical value, the grading of balloon cells in the asphyxia group is markedly elevated in comparison to the control group (*p* = 0.0239), whereas a marginal difference is noted in the grades of apoptotic cells (*p* = 0.0606) that was not statistically significant nevertheless. No difference was traced in the cases of stromal cells and vacuoles in oocytes.

**Figure 5:**
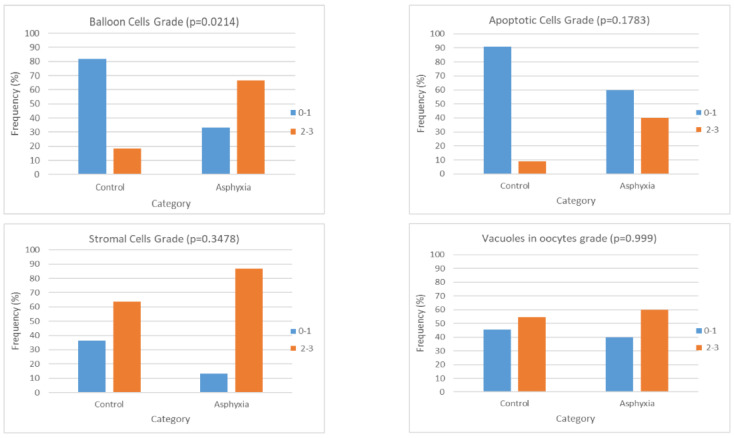
Charts presenting the frequency of histological gradings (classified as low: 0–1 or high: 2–3) in each of the study groups after categorizing them with respect to the application or not of asphyxia. Each chart is referring to one histological parameter (balloon cells, apoptosis, stromal cells, and vacuoles in oocytes), and the frequency is expressed as a percentage of the respective cases over the total cases of the specific group—control or asphyxia.

Moreover, a statistical analysis was performed on our data after categorizing them both with regards to the grading (0–1 vs. 2–3) and with regards to the application or not of asphyxia on the animals. According to the results, as far as the balloon cells are concerned, there was a marked differentiation of the cases with the higher grading (2–3) in the asphyxia group (with or without the application of resuscitation) (*p* value: 0.0214, OR: 9, 95% CI: 1.39–58.4). Therefore, it can be concluded that the presence of asphyxia can be considered a risk factor for the more prominent presentation of balloon cells. This is presented in Table 4 and depicted briefly in Figure 5.

Furthermore, we attempted to extract the Spearman’s correlation coefficients for the different histological parameters in pairs. The results as well as the respective *p* values (second line) are depicted in Table 5. There appears to be a moderate degree of correlation (r ≈ 0.4, *p* < 0.05) between the histological parameters of balloon cells and apoptosis, balloon cells with vacuoles in oocytes, and apoptosis with vacuoles in oocytes, whereas no correlation was noted between balloon cell grade and stromal cell grade, apoptosis and stromal cell grade, and stromal cell grade and vacuoles in oocytes grade.

In addition to the abovementioned analysis, we attempted to investigate the potential effect of the duration of asphyxia and resuscitation on the presence of the histological findings. Given that the timepoint of asphyxia was defined based on clinical criteria (presence of cardiovascular compromise), there was a large variation in the duration of asphyxia that appeared to range between 1.25 min and 28 min in the case of our study. In the cases where resuscitation was applied, the effect was assessed after every cycle of 30 s as indicated by the ILCOR guidelines, and depending on the outcome, resuscitation was continued or ceased. Consequently, the duration of resuscitation ranged from 1 to 2 min. The findings of this analysis were indicative of the presence of a statistically significant negative correlation between the duration of asphyxia and the grade of the presence of vacuoles in oocytes. Therefore, it appears that the longer duration of asphyxia inhibited the formation of vacuoles. This is presented in Table 6.

## 4. Discussion

### 4.1. Short- and Long-Term Effects of Prenatal and Perinatal Conditions on the Ovarian Function

To the best of our knowledge, no similar study has been performed until now. Therefore, although there is a huge amount of data in the literature on the effect of the prenatal and perinatal conditions on ovarian function and female fertility, there is no available information regarding the impact of an acute incident such as perinatal asphyxia. The multisystemic character and complications of perinatal asphyxia of the neonate, which are well described, could suggest that possible participation of the ovaries in this entity might be markedly reasonable. In the present study, we attempted to unveil potential alterations of the ovarian structure, on a histological basis, caused by asphyxia and resuscitation.

This hypothesis is further supported by the fact that ovarian function is affected by perinatal parameters, as reported in several studies mainly looking into the pathogenesis of premature ovarian failure (POF). Sadrzadeh et al. in a retrospective study reported that prematurity is a risk factor for premature ovarian insufficiency (POI) [14]. Cresswell et al. [15] suggested that poor growth and development in late gestation, manifested as smaller length at birth, leads to a smaller peak number of primordial follicles, which in turn may be linked to early menopause.

The correlation between age at menopause and BW standardized for gestational age, used as a marker of fetal environment and development, was also investigated in the 1958 British birth cohort study by Tom et al. [16], who reported that extremes of BW and higher BW standardized for gestational age were associated with earlier age at menopause.

In addition to this, there are several studies that are investigating the impact of intrauterine growth restriction (IUGR) on the ovarian development with reference not only to the ovarian histology but also to the ovarian function.

More specifically, Bruin et al. reported that the volume percentages of primordial follicles in the ovaries of growth-restricted fetuses were lower compared to age-matched controls with normal BW [17], thus suggesting that IUGR leads to a reduction in primordial follicle pool at birth. Alteration of follicle numbers as well as several genes’ expression in the ovary of IUGR offspring was also described in an animal study in rodents, which, however, highlighted the recovery of follicle number in peripubertal ages [18].

With regards to the female reproductive system, low BW as a consequence of intrauterine ‘malnutrition’ was also described to affect several parameters of puberty, such as the age of onset, the velocity of progression [19], the age of menarche, and the ovulation rate [20,21]. Girls born small for gestational age had increased levels of FSH both in infancy and adolescence [22,23], and this was interpreted by the authors as a form of ovarian hyporesponsiveness to FSH, possibly as a result of a diminished fraction of granulosa cells that is reminiscent in a way of reproductive aging.

In animal models, maternal undernutrition during pregnancy affected the fetal ovarian development in the form of a reduced number of primordial and secondary follicles, as well as alteration of the expression of genes that are regulatory for follicle maturation and ovulation [24]. Maternal food restriction caused a delay in fetal ovarian development, and interestingly, the findings were independent of effects on fetal mass [25].

Since IUGR is a condition that reflects chronic fetal tissue hypoxia, its effect on the female reproductive system could further support the hypothesis that perinatal asphyxia and the subsequent tissue hypoxia may also affect ovarian function in a similar way.

Several more studies attempted to find associations between in utero or early life parameters and age at menopause, based on the hypothesis that early life events may affect the initial follicular pool, as well as the rate of follicular loss and subsequently the age of menopause. Some of the factors that were looked into include in utero diethylstilbestrol exposure, maternal diabetes, and multiple pregnancy [26]. Finally, perinatal nicotine exposure also affected the fertility of the offspring in animal studies, and oxidative stress, inflammation, and endoplasmic reticulum stress were suggested as the pathology mechanisms of this effect [27].

While in utero conditions (resulting mainly in IUGR) were reported to affect the female reproductive system in several ways, the effect of acute situations in the perinatal period, such as perinatal hypoxia, is markedly less studied. However, it is reasonable to speculate that incidents and situations occurring prenatally or in the early life may affect ovarian function, as this is the case with other organs and systems.

Gutzeit et al. have recently studied the effect of hypoxic conditions imposed in the early postpartum period on the histology of the ovaries of female rat pups, drawing the conclusion that perinatal asphyxia leads to premature activation of dormant follicles and diminished ovarian reserve as well as decreased stromal cell proliferation [28].

In an attempt to investigate the effect of intrafollicular circumstances on the developmental capacity of human oocytes and taking into consideration the fact that each follicle has unique blood supply characteristics and perifollicular vasculature development, van Blerkom highlighted the fact that oocytes with aberrant organization and quality defects are relatively more common among populations of follicles that are more poorly vascularized. Furthermore, the conditions in the under-vascularized follicles were considered “severely hypoxic”, and the levels of oxygenation of follicular fluid were suggested to be related to the degree of perifollicular vasculature development [29]. Therefore, the speculation that hypoxic conditions during the oocyte maturation can lead to abnormalities and potentially affect the outcome in terms of fertilization and quality of the fetus can support the hypothesis of our study and implicate that hypoxia in an acute form (as in the cases of asphyxia) can be expected to affect the quality and development of the oocytes.

In their experimental study in rats, Xiao et al. highlighted the gender-dependent effect of antenatal hypoxia on the blood pressure response in adult offspring, suggesting a complex mechanism of programming of the cardiovascular system by antenatal hypoxia mediated through programming of the ovarian function [30]. The uncompromised ovarian function during development was also reported to be protective of the late effects of perinatal asphyxia on pulmonary vasculature in experimental studies in rats, which also highlighted the different sensitivities of pulmonary vessels to perinatal hypoxia between males and females [31].

### 4.2. Apoptosis

Oxender et al. [32] studied ovarian follicular development and quantified it in Dutch Landrace crossbred pigs aged 49 days postcoitum to 90 days after birth. The majority of the follicular population during late gestation and up until 90 days after birth consisted of primordial follicles. The number of germ cells was approximately 1,100,000 at 50 days postcoitum, declining to approximately 500,000 at birth [33] and further down to 420,000 by puberty [34].

Sheng et al. investigated the location of oocyte-specific linker histones at different developmental stages postpartum and reported that the histology of healthy newborn piglet ovaries at 3 days postpartum was mainly characterized by oogonia proliferation. Oogonia, egg nests, and synplasma-like cell groups constituted the ovary cortex, while syncytium-like cell groups and follicles were found in the interior of the cortex and medulla of the ovary [34]. Oogonia apoptosis was also noted in all of the ovaries, as was follicular atresia, and therefore, apoptosis is considered a common phenomenon in follicular development. This was also evident in our study, since apoptosis was present not only in the groups of animals that were asphyxiated (B and C) but also in the control group. In the same study and at a later phase of development (40–60 days postpartum), several vacuolated lipid droplets were highlighted in the follicular ooplasm with gradually increasing number and volume as the follicles grew in size. In primordial follicles undergoing atresia, oocytes became vacuolized in addition to other alterations. Further maturation of the ovaries at 72 to 165 days postpartum was characterized by the increase in stroma in the cortex among other changes, while the ooplasm lipid droplets became larger.

As mentioned above, apoptosis (which is a form of programmed cell death) was suggested as an ontogeny mechanism in the embryology of the mammalian ovaries. Given the significantly higher number of germ cells during embryonic life compared to the amount that actually evolve to form oogonia and further on develop to form follicles and reach ovulation, it can be speculated that a large number of germ cells are actually eliminated from the cohort of the ovary [35]. The suggested mechanisms of this procedure are apoptosis and autophagy, both leading to follicular atresia [36,37]. Apoptosis in the oocytes was described at all stages of oogenesis, and the morphological characteristics of apoptotic cells include cell shrinkage, nuclear condensation, membrane blebbing, and formation of apoptotic bodies [37]. As far as the mechanisms of apoptosis are concerned, they can be classified as intrinsic (mitochondria–mediated) or extrinsic (death-receptor mediated), depending on the biochemical pathways that occur, which can also intermediately interact [38]. Although the process of programmed cell death in the germ line has not entirely been deciphered so far, oxidative stress seems to be a significant factor augmenting the procedure of apoptosis through interaction with the intrinsic pathway [36]. Therefore, taking into consideration that both ischemia and ischemia/reperfusion may be causal factors of oxidative stress [39], it would be reasonable to expect that, in cases of perinatal asphyxia and subsequent oxidative stress, the apoptosis noted in the histological samples would be more marked. In the case of our study, the initial statistical analysis that included all the sample groups and all the grading values separately highlighted merely the presence of a trend of differentiation in the extent of apoptosis in the ovarian samples of piglets that had undergone asphyxia that was more prominent in the cases of animals that had been offered resuscitation as well. Nevertheless, when the categorization of the gradings was applied, there was a statistically significant difference that occurred. This could be indicative of the augmentation of the mechanism of apoptosis following asphyxia that appears to be further amplified in the cases of resuscitation, as the higher histological gradings appear to be more frequent in group C animal samples. This comes in accordance with our expectations based on pathophysiology as mentioned above since reoxygenation following asphyxia is considered to be an established factor leading to oxidative stress, and the latter has been shown to trigger apoptosis. Given the fact that apoptosis as well as other pathology lesions might be more prominent as time progresses following an insult, we can speculate that if our study’s design allowed for a more prolonged period of observation following asphyxia and reoxygenation (in the cases where it was offered), the histological lesions might have been more outstanding. In addition to this, perhaps had the group population been bigger, there would have been a more prominent difference noted.

Apoptosis was also described as a pathophysiological mechanism of ovarian damage caused by environmental factors or industrial elements such as 4-vinylcyclohexene diepoxide [40]. Halicioglu et al. looked into the effect of 4-vinylcyclohexene diepoxide on the ovaries, and vacuolization and increased collagen fibers both in the cortex and medulla of the ovaries were considered additional histopathological lesions indicative of ovarian damage [40].

### 4.3. Vacuoles in Oocytes

Vacuoles in oocytes are, in general, considered an indication of severe degeneration and one of the parameters that are frequently assessed when considering oocytes as dysmorphic [41]. According to the available literature, the vacuoles vary in size and can be present in 5% to 12% of the oocytes [41]. Vacuoles in oocytes were also considered (among others) as markers of ovarian degeneration in rodents in an experimental study by Ural et al., who looked into the biochemical, histopathological, and immunohistochemical changes in adult female rats undergoing ovarian torsion, as well as the potential therapeutic effect of thymoquinone administration, expected to act as an antioxidant factor. Vacuolization of the oocyte cytoplasm was more prominent in the animals exposed to hypoxia (ovarian torsion) and particularly more prominent in the cases where reoxygenation was applied. However, the extent of the lesions was alleviated in the cases where thymoquinone was administered [11]. With regards to its clinical impact, oocyte vacuolization (considered a cytoplasmic abnormality during oocyte maturation) was also noted to be relatively more frequent in patients with infertility undergoing IVF [42,43]; in terms of pathophysiology, it was attributed to either uncontrollable endocytosis or fusion of vesicles produced by the smooth endoplasmic reticulum and Golgi apparatus that failed to be exocytosed [42], since the fluid within the vacuoles was identical to the fluid within the perivitelline space in the study of Van Blerkom et al. [44]. Alternative theories suggest that the vacuoles may occur as a result of dilation of saccules of the smooth endoplasmic reticulum (SER) or be attributed to some form of instability in the organization of the cortical cytoplasm [44]. Vacuoles in oocytes have also been found to be associated with lysosomes [45] or actin cytoskeleton dysfunction [46]. However, the origin of the morphological abnormalities of the oocytes has not been clarified and is anticipated to be multifactorial. Both intrinsic and extrinsic factors are speculated to contribute to their formation [41], and the extent to which oxidative stress, inflammation, or hypoxia might affect their presence is not known [46]. Existing data in the literature suggest that oocyte vacuolization may be considered a dysmorphic feature indicative of poor maturation of the cytoplasm that can lead to both fertilization failure and early pregnancy loss [47]. However, the data referring to the clinical implications of the oocyte vacuoles are controversial. According to several studies, both the fertilization potential of the oocytes and the quality and developmental capacity of the embryo in case of fertilization are reported to be affected negatively by the presence of vacuoles, but these findings are not consistent in all of the studies and cases [41,45,46]. Therefore, more research is required in unveiling the significance and impact of their presence.

It was fairly interesting that in our study there was a statistically significant negative correlation between the grade of vacuoles and the duration of asphyxia. This could partly account for the absence of a difference in the presence of vacuoles between the different study groups through masking the results. More specifically, based on the fact that vacuolization is considered a sign of degeneration, we would expect it to be more prominent in the samples from the animals that had been exposed to asphyxia. Nevertheless, asphyxia might have a more complicated effect on the formation of the vacuoles, and if prolonged, it might inhibit it, thus alleviating their overall incidence in the asphyxiated animals. Since the pathophysiology and exact mechanisms of their creation are not fully understood, it is more challenging to explicate the above finding. Perhaps it can be partly attributed to the energy and oxygen deprivation that characterizes asphyxia that could impair the fusion of vesicles or the process of endocytosis if we consider that these mechanisms lead to the vacuole formation. In a study that was conducted by Van Blerkom that mainly referred to the period of oocyte maturation around the first polar body abstriction, vacuoles appeared to form rapidly in time periods as short as a few minutes occasionally, and the whole process is considered to be dynamic since they can also fragment or combine and merge [44]. Therefore, we could speculate that in the case of our study, the time intervals that were applied before the removal of the ovaries could be sufficient for alterations in the frequency and presence of vacuoles, either in the form of augmentation or in that of reduction. If the impact of asphyxia on the formation of oocyte vacuoles is multidimensional, the outcome might be justifiably indifferent to the control group.

### 4.4. The Role of Stroma

The role and alterations/variations of the ovarian stroma function have not been completely decrypted so far. In a nomenclature for proliferative and nonproliferative lesions in laboratory animals that was generated and published as part of the INHAND Project, interstitial cell hypertrophy and interstitial cell hyperplasia are described as histological changes that are commonly observed in ovarian aging and atrophy in adult rodents. They can also be encountered in cases of gonadotropin administration or exposure to xenobiotics such as organophosphate and thiocarbamate compounds. Sex cord stromal hyperplasia is also regarded as a common lesion in old rats [48]. The ovarian stroma, a term referring to the components of the ovary that are not ovarian follicles, consists of a variety of cell types, some subtypes of which have not been fully characterized so far, such as immune cells, blood and lymphatic vessels, ovarian surface epithelium, hilar cells and stromal cells, and ovarian extracellular matrix components [49]. The role of the stroma is highly supportive and very significant for the follicular development and maturation. Among other alterations, ovarian stromal hyperplasia is observed in cases of polycystic ovary syndrome (PCOS) [49]. Additional alterations in the ovarian stroma reported to occur in cases of aging or oxidative stress (which would be expected to be present in cases of ischemia and reoxygenation) that were, however, not investigated in our study, include fibrosis and inflammation that lead to the disruption of the microenvironment of the follicles, subsequently affecting the gametes quality and quantity [50].

### 4.5. Balloon Cells

As far as balloon cells are concerned, ballooning degeneration is a histological alteration of cells characterized as a form of cellular edema. It has been mainly described in hepatocytes and is present in several types of hepatic conditions, more commonly encountered in fatty liver disease [51]. In an experimental study investigating the characteristics of the refeeding syndrome in rats, ballooning degeneration was the main histological characteristic in the group of rodents that had been exposed to fasting for 48 h followed by refeeding for more 48 h [52]. In the same study, this lesion was the outcome of impairment of cell volume regulation caused by malfunction of the Na^+^/K^+^ pump of the cellular membrane. Since the role and function of this pump is to actively excrete Na^+^ from the cells into the extracellular fluid and allow influx of K^+^ into the cell against the osmotic gradient, it can be concluded that in cases of malfunction, there is entry of Na^+^ followed by water into the intracellular space, thus leading to cellular edema. This malfunction can present in cases of hypoxia, lack of ATP, oxidation, and degradation of enzymes or infectious conditions [52]. An alternative hypothesis for the pathophysiology of ballooning degeneration of hepatocytes suggests that it can be attributed to alteration of the intermediate filament cytoskeleton [51]. In an experimental model aiming at unveiling the mechanisms regulating the life cycle of the ovarian cells and the potential effect of local trophic factors and their inhibitors, such as acetylcholine (Ach) and acetylcholinesterase (AChE), the authors referred to the presence of necroptosis, defined as a type of programmed necrosis and characterized by a balloon-like morphology of the cells [53]. In the case of our study, during the initial statistical processing, there is a marked trend of increasing histological grading with regards to balloon cells between the cases of asphyxiated and control piglets, and this trend is further extended to the group of piglets that were offered resuscitation following asphyxia. When categorization is applied in the grading and it is assessed as either low (0–1) or high (2–3), there is the occurrence of a statistically significant difference between the animals that underwent asphyxia (either with or without resuscitation) and the control animals, indicating that ballooning degeneration can be considered a direct effect of asphyxia in the ovarian histology. Furthermore, taking into consideration the limitation of the relatively small amount of samples, given the fact that there is no marked difference between the samples of the animals that had been offered resuscitation and the ones that had not, it can be speculated that this lesion is possibly not reversible, at least within the proximate time period following return of spontaneous circulation. Further categorization of our samples with regards to the application or no of asphyxia (irrespective of resuscitation) either in combination with grading categorization (as previously mentioned) or without proved to support the abovementioned conclusion regarding the ballooning degeneration of the oocytes occurring as a result of asphyxia.

### 4.6. Limitations and Strengths of This Study

Although no differentiation was noted with regards to vacuoles in oocytes and the presence of stromal cells, the findings in reference to balloon cells and apoptosis could indicate that perinatal hypoxia and resuscitation might indeed affect the ovarian function and histology, and perhaps if the population of the animals was larger additional statistically significant differences could be highlighted, and further conclusions could be drawn regarding the extent to which the histological markers that we investigated could be present regardless of the application of hypoxia and reoxygenation and therefore constitute baseline traits of the ovarian histology subject to individual variability. With a larger sample size, it is likely that more subtle yet significant variations in ovarian histology could have been highlighted, allowing for more robust conclusions regarding the extent to which the histological markers we evaluated might be present, regardless of the application of hypoxia and reoxygenation. Additionally, a larger sample size would have allowed us to better assess the extent to which individual variability influences these baseline histological characteristics, potentially unveiling underlying patterns in ovarian development and response that were not entirely identified with the current sample size.

An additional impression that was created from the pathology examination was that in some of the samples there are histological lesions only in the cortex (where the follicles are located), while there are no apparent alterations in the medulla histology. Given that the medulla is highly vascular, it could be speculated that in the cases of mild hypoxia, the medulla is expected to be more “resistant” compared to the cortex, and this speculation is consistent with our observation.

The fact that during the experimental procedure, some of the animals failed to meet the necessary criteria to be admitted to this study can be considered a limitation of our study. If we had managed to obtain and process valid tissue samples from 33 animals as was originally planned in our study, we might have succeeded in highlighting the presence of statistically significant findings with regards to all of the histological parameters that were assessed. However, taking into consideration the 3 Rs principle in laboratory animal experimentation (Replacement, Reduction, Refinement) [54] and the fact that statistically significant results occurred despite the reduced number of study animals, it was considered inappropriate to include more animals in our study through continuing the experiments. In contrast, the strength of our study was considered to be amplified.

An additional limitation of our study is the short time of observation and the fact that our experiment is acute. More specifically, according to the design of our study in the cases of the animals that were exposed to asphyxia with no resuscitation, the ovarian tissues were retrieved directly after the manifestation of cardiovascular compromise and asphyxia and prior to the animals’ death, while in the cases of the animals that were resuscitated, an additional time period of 30 min of observation following ROSC was provided before harvesting of the ovarian tissues. The reasons for these short time periods are technical and ethical since the fierce application of highly hypoxic conditions (aimed at simulating an episode of perinatal asphyxia) would be expected to severely affect the animals’ well-being and cause them discomfort that was not considered appropriate to be extended in the cases of the animals that were resuscitated, while in the cases of the animals that were not resuscitated, it would be technically impossible to extend the period of observation.

The power analysis of our study was performed as already mentioned based on the study by Ural et al. [11] that investigated the effect of ovarian torsion on the histology and function of the ovaries of adult rodents as well as the reversibility of the lesions following the application of an antioxidant factor. The limitations of this extrapolation are mainly related to the different duration of asphyxia and reoxygenation applied since the respective periods of time were defined as 3 h in the case of the abovementioned study compared to 30 min of observation following reoxygenation (in the cases where resuscitation was applied) and removal of the ovaries directly at the point of asphyxia in the cases where resuscitation was not offered. Furthermore, the experimental study run by Ural et al. was applied to adult animals rather than neonates, as was the case in our study. Similar time intervals (3 h of ischemia and 3 h of reperfusion) were maintained in additional experimental models [55]. It would be worth investigating whether prolonged ischemia and the extended period of observation post-resuscitation-reperfusion would markedly affect the ovarian histology of newborn piglets. This limitation could be expected to affect the external validity of our study and the applicability of the results to humans, since in cases of perinatal asphyxia or stress that does not lead to the newborn’s demise, the period of “observation” is further extended to more than 30 min, and additional factors such as treatment modalities and medication that is administered might further affect the outcome and effect on the ovaries either in a protective or in an aggravating way. In addition to this, in the cases of the neonates that reach puberty, functional alterations could manifest that might not be reflected in the histological appearance of the tissues.

In the absence of similar studies, it remains fairly challenging to determine the precise time required for histological changes to occur in ovarian tissue, particularly in neonatal models. While some studies have investigated longer observation periods for ovarian pathology in other contexts, the acute and severe nature of our experimental conditions significantly limits the ability to determine the exact time frame needed for histological lesions to manifest. Thus, although the short observation period is a limitation, it was necessary in order to balance the need for scientific insight with the ethical obligation to protect the animals from unnecessary distress and suffering. This limitation should be taken into consideration when interpreting the findings, as the full scope of histological changes in response to perinatal asphyxia may require longer exposure times than the ones that were reached in the frame of our study.

Another factor that might influence the external validity of our study is the experimental nature itself and the fact that it was performed on a different species. However, the design of our study could not have been applied to human neonates for ethical reasons, and as already mentioned, swine are considered to be a species bearing close resemblance to humans with regards to anatomy, histology, and physiology, and therefore, we would expect that the findings of our study can be extrapolated to humans to some degree.

Nevertheless, apart from factors related to the experimental design, an additional reason that could contribute to the absence of statistically significant results in our study with regards to some of the histological lesions that were looked into could be that prepubertal ovaries were less susceptible to the effects of both gonadotoxic medication and hypoxia [56]. More specifically, with regards to hypoxia, in a recent study attempting to investigate whether age plays a significant role in the hypoxia-induced damage to the ovarian reserve, hypoxic damage was found to lead to diminished ovarian reserve in an age-dependent manner, with adult ovaries noted to be markedly more susceptible compared to newborn ovaries. In the same study, the ovarian reserve depletion caused by hypoxia was attributed to premature activation and initiation of growth in dormant follicles, leading to ovarian follicular “burnout”, rather than to an increase in apoptosis [56]. Therefore, newborn ovaries might have “defense mechanisms” against hypoxia or the absence of initiation of function itself during neonatal life, and the subsequent decrease in the oxygen and energy requirements might act as a protective mechanism against hypoxia.

Moreover, hypoxia is reported to be a common state for the growing follicles [57,58] since the thecal capillaries do not penetrate the membrana propria, and the granulosa layer remains avascular during the follicular development. The subsequent state of hypoxia in the follicular fluid leads to increased VEGF expression from the granulosa cells, which is essential for the development of a rich vascular network within the follicular wall, while the oocyte development has been found in experimental studies to be greatly affected by the presence of certain growth factors even in hypoxic conditions, which are resembling in vivo circumstances [59]. Therefore, it is suggested from experimental studies that large swine follicles are physiologically exposed to a state of oxygen deprivation that acts as a stimulator for the development of the ovarian vasculature [60]. Therefore, it could be speculated that the ovarian tissue is developmentally prepared to encounter hypoxia, and this could lead to the alleviation of the pathological effects in cases of asphyxia.

Last but not least, on top of the differences in the ovarian histology that were highlighted in our study between the animals that underwent perinatal asphyxia with or without resuscitation and the control animals, an interesting conclusion can be drawn regarding the baseline histology of the ovaries of neonate female piglets, and potentially further assumptions can be made regarding the embryology of the ovarian development in mammals.

### 4.7. Future Considerations

Although the findings of our study support the hypothesis that the ovarian histology is affected and altered as a result of perinatal asphyxia, it would be very interesting and useful to attempt to correlate these histological alterations with potential functional disturbances in the future. Perhaps a study of a different design that could allow for the long-term observation of the study subjects could highlight the long-term impact of perinatal asphyxia on fertility or other aspects of the ovarian systemic effects. In addition to this, perhaps a study looking concomitantly at the histological lesions of the ovaries and the cardiovascular adaptations to perinatal asphyxia or the CNS development following an acute incidence could manage to further clarify the origins of the sex-related effects of asphyxia on neonates and the extent to which it could be mediated by disruptions of the ovarian histology and function.

Furthermore, in a non-experimental context, a study aiming at following up infants that underwent perinatal hypoxia/asphyxia in adult life might facilitate uncovering the potential existence of a correlation between entities of the female adult life referring to the reproductive or cardiovascular system and incidents of the perinatal life in the form of hypoxia/asphyxia. This could be applied not only in better understanding the pathophysiology of certain diseases and conditions that are not fully comprehensible but also in offering better management policies to the patients.

## 5. Conclusions

In our study, we managed to unveil the presence of certain histological alterations (balloon cells and apoptosis) at a more extended degree in the cases of the animals that underwent perinatal asphyxia. Furthermore, as far as apoptosis is concerned, there was also a statistically significant predominance in the cases of animals that were resuscitated following asphyxia. However, it would be worth considering and further investigating the potential existence of protective mechanisms that limit the hypoxic damage in the ovaries, which could partly account for the absence of significant findings with regards to the rest of the parameters that were investigated in the case of our study.

Further research is needed in order to clarify the effect of perinatal hypoxia and asphyxia on the ovaries, and in the long term, this could also facilitate the decryption of pathophysiological mechanisms implicated in the ovarian function and development.

## Figures and Tables

**Table 1:** Baseline characteristics of the study groups.

	A (CTRL) (N = 11)	B (ASPH) (N = 9)	C (RESC) (N = 6)	*p*-Value
Age (days)	2 (1, 4)	2 (1, 3.5)	2 (1.75, 2.5)	0.778 kw
Weight (grams)	1540 (1400, 1700)	1500 (1337.5, 1735)	1530 (1405, 1665)	0.791 kw
Baseline pH	7.34 ± 0.0944	7.41 ± 0.0746	7.37 ± 0.0468	0.224 av
Baseline Lac (mmol/L)	0.64 (0.53, 1.14)	0.91 (0.425, 1.00)	1.03 (0.55, 1.33)	0.62 kw
Baseline HR (bpm)	151 ± 36.2	139 ± 21.9	148 ± 26.1	0.694 av
Baseline MAP (mmHg)	61.0 ± 13.5	54.3 ± 9.76	46.5 ± 10.3	0.0772 av
Baseline SpO_2_	0.969 ± 0.0273	0.960 ± 0.0224	0.967 ± 0.0273	0.74 av

Abbreviations: CTRL: control, ASPH: asphyxia, RESC: resuscitation, Lac: lactate, HR: heart rate, MAP: mean arterial pressure, SpO_2_: oxygen saturation. Data are presented as median values. Within square brackets are reported the quartile 1 and quartile 3. kw: Kruskal–Wallis test, av: ANOVA test.

**Table 2:** Descriptive statistics of the histological grading per animal group.

Category	N	Label	Mean	SD	Median	Q1	Q3
**A (CTRL)**	11	**Balloon Cells Grade**	0.91	0.7	1	0	1
		**Apoptotic Cells Grade**	0.36	0.67	0	0	1
		**Stromal Cells Grade**	1.73	1.42	2	0	3
		**Vacuoles in oocytes grade**	1.82	0.87	2	1	3
**B (ASPH)**	9	**Balloon Cells Grade**	1.56	1.01	2	1	2
		**Apoptotic Cells Grade**	0.89	1.27	0	0	1
		**Stromal Cells Grade**	2.11	1.27	3	2	3
		**Vacuoles in oocytes grade**	1.67	1.12	1	1	3
**C (RESC)**	6	**Balloon Cells Grade**	2	0.89	2	1	3
		**Apoptotic Cells Grade**	1.67	1.03	2	1	2
		**Stromal Cells Grade**	2.83	0.41	3	3	3
		**Vacuoles in oocytes grade**	2.17	0.75	2	2	3

Abbreviations: Group A (CTRL): control, Group B (ASPH): asphyxia, Group C (RESC): asphyxia and resuscitation, SD: standard deviation, Q1: first quartile, Q3: third quartile.

**Table 3:** Histological gradings for the 4 parameters referring to each of the 3 groups of animals using a classification of the gradings (0–1 and 2–3).

**Category**	**Balloon Cells Grade (*p* = 0.04871, x^2^ = 6.0)**			**Category**	**Apoptotic Cells Grade (*p* =0.0364, x^2^ = 6.7)**		
	**0–1**	**2–3**	**Total**		**0–1**	**2–3**	**Total**
**A (CTRL)**	9	2	11	**A (CTRL)**	10	1	11
	81.82	18.18	100		90.91	9.09	100
**B (ASPH)**	3	6	9	**B (ASPH)**	7	2	9
	33.33	66.67	100		77.78	22.22	100
**C (RESC)**	2	4	6	**C (RESC)**	2	4	6
	33.33	66.67	100		33.33	66.67	100
**Total**	14	12	26	**Total**	19	7	26
	53.85	46.15	100		73.08	26.92	100
**Category**	**Stromal Cells Grade (*p* = 0.3012** **, x^2^ = 2.9)**			**Category**	**Vacuoles in oocytes grade (*p* = 0.4152** **, x^2^ = 2.3)**		
	**0–1**	**2–3**	**Total**		**0–1**	**2–3**	**Total**
**A (CTRL)**	4	7	11	**A (CTRL)**	5	6	11
	36.36	63.64	100		45.45	54.55	100
**B (ASPH)**	2	7	9	**B (ASPH)**	5	4	9
	22.22	77.78	100		55.56	44.44	100
**C (RESC)**	0	6	6	**C (RESC)**	1	5	6
	0	100	100		16.67	83.33	100
**Total**	6	20	26	**Total**	11	15	26
	23.08	76.92	100		42.31	57.69	100

Abbreviations: A (CTRL): control group, B (ASPH): asphyxia group, C (RESC): asphyxia and resuscitation group. In each cell, there is a presentation of the number of cases in each category (1st line: numerical number of cases per category, 2nd line: percentage of the respective cases over the total cases of the specific group—A, B, and C).

**Table 4:** Histological grading (classified as either low 0–1 or high 2–3) in correlation with the study groups classified with regards to the application or not of asphyxia.

**Category**	**Balloon Cells Grade (*p* = 0.0214, x^2^ = 6.0)**			**Category**	**Apoptotic Cells Grade (*p* = 0.1783, x^2^ = 3.1)**		
	**0–1**	**2–3**	**Total**		**0–1**	**2–3**	**Total**
**Control**	9	2	11	**Control**	10	1	11
	81.82	18.18	100		90.91	9.09	100
**Asphyxia**	5	10	15	**Asphyxia**	9	6	15
	33.33	66.67	100		60	40	100
**Total**	14	12	26	**Total**	19	7	26
	53.85	46.15	100		73.08	26.92	100
**Category**	**Stromal Cells Grade (*p* = 0.3478, x^2^ = 1.9)**			**Category**	**Vacuoles in oocytes grade (*p* = 0.999, x^2^ = 0.08)**		
	**0–1**	**2–3**	**Total**		**0–1**	**2–3**	**Total**
**Control**	4	7	11	**Control**	5	6	11
	36.36	63.64	100		45.45	54.55	100
**Asphyxia**	2	13	15	**Asphyxia**	6	9	15
	13.33	86.67	100		40	60	100
**Total**	6	20	26	**Total**	11	15	26
	23.08	76.92	100		42.31	57.69	100

Footer: In each cell, there is a presentation of the number of cases in each category (first line: numerical number of cases per category, and second line: percentage of the respective cases over the total cases of the specific group—control or asphyxia).

**Table 5:** Correlation coefficients between the histological parameters and respective p values.

		Apoptotic Cells Grade	Stromal Cells Grade	Vacuoles in Oocytes Grade
Balloon Cells Grade	r	0.46975	0.17344	0.46318
	*p* value	0.0155	0.3968	0.0172
Apoptotic Cells Grade	r		0.28307	0.43086
	*p* value		0.1611	0.028
Stromal Cells Grade	r			0.32885
	*p* value			0.1009

**Table 6:** Correlation coefficients (rs) between the duration of asphyxia and the grading of the histological parameters and duration of resuscitation.

Variable	Measure	*p*-Value, Ν
Category	B (ASPH): 14 (1.2–28), C (RESC): 16 (1.5–20)	*p* = 0.906, N = 15
Balloon cells grade	rs = −0.24	*p* = 0.384, N = 15
Apoptotic cells grade	rs = −0.25	*p* = 0.359, N = 15
Stromal cells grade	rs = 0.17	*p* = 0.541, N = 15
Vacuoles in oocytes grade	rs = −0.54	***p* = 0.039**, N = 15
Duration of resuscitation (minutes)	rs = −0.15	*p* = 0.774, N = 6

Abbreviations: ASPH: asphyxia, RESC: resuscitation. N: number of samples available for assessment.

## Data Availability

The raw data supporting the conclusions of this article are available by the corresponding authors on request.
